# Cell cycle arrest by a gradient of Dpp signaling during Drosophila eye development

**DOI:** 10.1186/1471-213X-10-28

**Published:** 2010-03-09

**Authors:** Lucy C Firth, Abhishek Bhattacharya, Nicholas E Baker

**Affiliations:** 1 Department of Molecular Genetics, Albert Einstein College of Medicine, 1300 Morris Park Avenue, Bronx, NY, 10461, USA; 2 Current address: Syngenta, Jealott's Hill International Research Center, Bracknell, Berkshire RG42 6EY, UK

## Abstract

**Background:**

The secreted morphogen Dpp plays important roles in spatial regulation of gene expression and cell cycle progression in the developing *Drosophila *eye. Dpp signaling is required for timely cell cycle arrest ahead of the morphogenetic furrow as a prelude to differentiation, and is also important for eye disc growth. The *dpp *gene is expressed at multiple locations in the eye imaginal disc, including the morphogenetic furrow that sweeps across the eye disc as differentiation initiates.

**Results:**

Studies of Brinker and Dad expression, and of Mad phosphorylation, establish that there is a gradient of Dpp signaling in the eye imaginal disc anterior to the morphogenetic furrow, predominantly in the anterior-posterior axis, and also Dpp signaling at the margins of the disc epithelium and in the dorsal peripodial membrane. Almost all signaling activity seems to spread through the plane of the epithelia, although peripodial epithelium cells can also respond to underlying disc cells. There is a graded requirement for Dpp signaling components for G1 arrest in the eye disc, with more stringent requirements further anteriorly where signaling is lower. The signaling level defines the cell cycle response, because elevated signaling through expression of an activated Thickveins receptor molecule arrested cells at more anterior locations. Very anterior regions of the eye disc were not arrested in response to activated receptor, however, and evidence is presented that expression of the Homothorax protein may contribute to this protection. By contrast to activated Thickveins, ectopic expression of processed Dpp leads to very high levels of Mad phosphorylation which appear to have non-physiological consequences.

**Conclusions:**

G1 arrest occurs at a threshold level of Dpp signaling within a morphogen gradient in the anterior eye. G1 arrest is specific for one competent domain in the eye disc, allowing Dpp signaling to promote growth at earlier developmental stages.

## Background

The BMP-class ligand Dpp can act as a graded morphogen during development. In the developing wing, a bi-directional Dpp gradient that spreads from its stable source near the A/P compartment boundary defines many aspects of anterior-posterior position for wing imaginal disc cells [1,2]. The related molecule Activin also acts as a graded morphogen in *Xenopus *development[3,4].

It is proposed that Dpp also functions as a gradient morphogen to pattern the anterior-posterior axis of the eye imaginal disc, but the progressive nature of eye development makes comparison to the wing disc complicated [5]. The eye differentiates asynchronously, as a 'morphogenetic furrow' moves across the eye imaginal disc from posterior to anterior [6]. Because Dpp is expressed in the morphogenetic furrow, this source moves across the eye disc as differentiation proceeds [7].

In eyes, the main evidence for a morphogen gradient of Dpp is that distinct effects of Dpp signaling are manifested at particular distances anterior to the MF. Although consistent with a Dpp morphogen gradient, other mechanisms could also explain this. When each particular response to Dpp is considered, it turns out that alternatives to the morphogen mechanism are plausible in every case. For example, Dpp-dependent genes are expressed in the anterior eye disc in distinct, overlapping expression domains. These domains might reflect activation at different thresholds in a Dpp gradient, but it is also possible that the differences reflect combinatorial interactions with other signaling pathways (see Discussion).

BMP signaling is also required for eye disc cells to arrest in G1 phase of the cell cycle. Cells normally arrest anterior to the morphogenetic furrow, as a prelude to cell fate specification and differentiation. Cells mutant for BMP receptors or signal transducers arrest significantly later than normal [8,9]. Therefore, G1 arrest may reflect regulation of an unidentified cell cycle target gene by Dpp signaling [10]. An alternative possibility relates to the control of growth by Dpp signaling, where 'growth' refers to cellular mass accumulation[11]. If cells that lack Dpp signaling components grow more slowly within the asynchronously-dividing, anterior portion of the eye disc, they might take longer to reach G1 after receiving a signal to arrest, and therefore arrest later than nearby wild type cells even if the timing and positioning of the arrest signal was not Dpp-dependent.

It has not been demonstrated directly that Dpp signaling is actually graded in the anterior-posterior axis of the eye disc. Dpp is transcribed not only in the MF, but also at the dorsal and ventral margins of the eye disc, close to the boundary between disc epithelium and peripodial epithelium[7]. A second BMP protein that might also interact with Dpp receptors, Gbb, is transcribed reciprocally to Dpp [12]. The *brinker (brk) *gene has been described as a further BMP target whose transcription is inhibited by BMP signaling [13-15]. It has been suggested that *brk *is the most direct transcriptional target of Dpp[16]. Unlike the other responses mentioned, *Brk-LacZ *reporter constructs are expressed in an equatorial-to-polar gradient, implying a polar-to-equatorial gradient of Dpp signaling[16]. Further evidence for a polar-to-equatorial gradient of Dpp signaling comes from the role of Dpp in competitive growth. Cells mutant for BMP receptors or signal transducers survive and proliferate more successfully close to the equator than close to the poles, consistent with a reduced requirement for BMP signaling for growth and survival close to the equator [8]. A additional complication is observations with Dpp-GFP fusion protein that suggest Dpp protein may spread abundantly through the imaginal disc lumen, contacting the apical surface of all eye disc cells approximately uniformly [17]. In addition to ligands, the spatial pattern of signaling might be affected by the multiple receptor species that are expressed, since both Type 1 receptors Sax and Tkv are required in the eye [8,9]. Thus, the actual distribution of BMP signals is uncertain.

Here, we report a detailed characterization of the role of Dpp signaling in establishing G1 arrest in a spatial domain of the eye disc. This is particularly interesting because of the evidence that Dpp signaling is required both for growth of eye disc cells and for their cell cycle arrest, two seemingly incompatible roles. Our findings strongly support a particular threshold of Dpp signaling within an anterior-posterior gradient as the trigger for G1 arrest, but also argue that many regions of the growing eye and antennal discs are protected from this response.

## Results

### BMP signaling activity in situ

**Figure 1:**
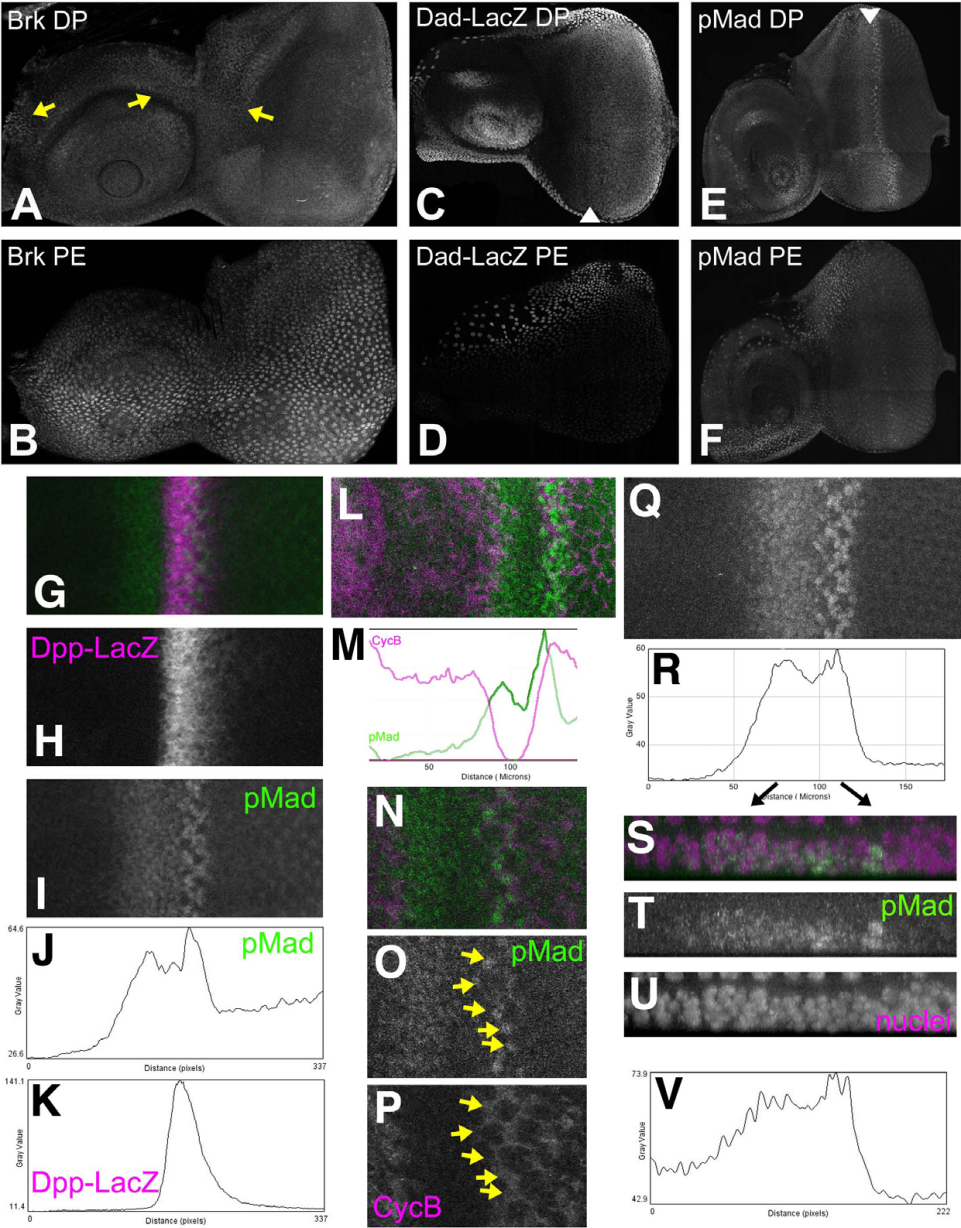
**Dpp signaling in the third instar eye-antennal disc**. Posterior is to the right and dorsal uppermost in all preparations. (A) Nuclear Brk protein was detected only in parts of the dorsal antennal disc and a few cells at the very anterior of the eye disc proper (arrows); (B) Nuclear Brk protein is strongly detected in peripodial membrane cells. There is a high-to-moderate gradient from ventral-to-dorsal; (C) A positive target of BMP signaling, DadLacZ, was active from 5-6 cell diameters anterior of the morphogenetic furrow and more posteriorly. Arrowhead indicates the morphogenetic furrow. Expression begins slightly earlier (more anteriorly) near the disc margin; (D) DadLacZ was detected in the peripodial epithelium over the ventral antennal disc; (E) Phosphorylated Mad protein is detected in nuclei in a broad domain centered on the MF, extending only slightly more anteriorly at the disc margin, and in a stripe of cells posterior to the furrow. Arrowhead indicates the morphogenetic furrow. Weak labeling of photoreceptor cells in the posterior of the disc is non-specific, since it is unaffected in Mad mutant cells (not shown); (F) Phosphorylated Mad is absent from the peripodial epithelium apart from a few cells dorsally; (G) The Dpp-LacZ transgene is expressed within the morphogenetic furrow (magenta), overlapping the domain of Mad phosphorylation (green). It is possible that Dpp-LacZ might lag behind endogenous Dpp protein; (H) Dpp-LacZ; (I) pMAd; (J) profile plot of the pMad labeling shown in panel I; (K) Profile plot of the Dpp-LacZ shown in panel H. (L) An eye disc labeled with phosphorylated Mad (green) and Cyclin B (magenta) shows that the cells posterior to the furrow where Phospho-Mad levels peak also express the Cyclin B associated with the SMW; (M) Profile plots of the CycB and pMad levels from panel L. (N) A close up of a single confocal z-plane, doubly labeled with CycB and pMad like that shown in panel L. Strongly pMad-positive cells are also labeled with CycB (arrows in panels O, P); (O) pMad labeling from panel N. (P) CycB labeling from panel N. (Q) pMad labeling of an eye disc; (R) Profile plot of the labeling shown in panel Q; (S) A 5 micron strip from panels Q & R, magnified and re-projected to show pMad labeling (green) from the side. Apical disc surface uppermost. Nuclei of all cells are labelled with DRAQ5 (magenta); (T) pMad labeling from panel S; (U) DRAQ5 labeling of all nuclei from panel S; (V) A profile plot from the lateral region of an eye imaginal disc.

Transcription of the *brk *gene is repressed by BMP signaling [13-15]. Because LacZ reporter patterns may lag behind actual transcription due to perdurance of the beta-Galactosidase protein, we visualized Brk protein directly. In antennal discs, Brk protein was detected in the nuclei of cells in the dorsal antennal disc, except at the anterior-posterior compartment border, approximately reciprocal to the transcription of Dpp just anterior to the compartment border, especially in the ventral antenna (Figure 1A). In eye discs, Brk protein was only detected in cells adjoining the dorsal anterior antenna, at the very anterior of the disc. Otherwise, no Brk was detected in much of the disc proper (Figure 1A). By contrast, robust Brk expression was easily detected in most cells of the peripodial epithelium overlying both eye and antennal discs, perhaps more strongly on the ventral side (Figure 1B). These findings indicate that nearly all eye disc epithelium cells experience BMP signaling activity, but little BMP signaling is occurring in the peripodial epithelium.

We also examined a DadLacZ reporter which is positively induced by BMP signaling. DadLacZ was active from 4-5 cell diameters anterior of the MF backwards to the posterior of the disc. DadLacZ extended slightly more anteriorly at the poles of the disc than at the equator (Figure 1C). DadLacZ confirms that there is BMP activity in the eye disc, and suggests a predominantly posterior-to-anterior distribution, with a minor polar-to-equatorial component in addition. The probable perdurance of beta-galactosidase makes it uncertain how far Dad transcription and BMP signaling continue posterior to the MF. DadLacZ was also detected on the dorsal side of the peripodial epithelium over the antennal disc (Figure 1D) in the region that contains lower Brk levels, consistent with Dpp signaling there.

To obtain a real time picture of BMP signaling, Mad phosphorylation was examined with the phospho-Mad (pMad) antibody[18]. In the eye disc, pMad levels accumulate in a broad band of cells anterior to and within the MF, terminating in a stripe of more intensely-labelled cells around columns 3 and 4 at the posterior edge of the furrow (Figure 1E) [19]. In antennal discs, anti-pMad labels a broad band of nuclei in the ventral region of the disc (Figure 1E). All of this labelling was reduced in cells mutant for *Mad*, confirming the specificity of the antibody (data not shown). Peripodial cells were positive for pMad over the ventral region of the antennal disc and the dorsal region of the eye disc (Figure 1F). In some preparations, nuclear pMad was also weakly detected in peripodial epithelium cells directly overlying the morphogenetic furrow, indicating some peripodial response to Dpp from the disk proper. Signals from the peripodial epithelium to the disc proper have been shown to be important for growth, patterning of the retinal epithelium and MF progression [20,21]. Dpp has also been observed in the lumen between the two epithelial layers [17]. The inter-epithelial signaling we observe over the morphogenetic furrow was in the opposite direction, and seemingly at a level too low to induce DadLacZ or repress Brk.

To determine whether there was a gradient of Mad phosphorylation, pMad was labelled in DppLacZ discs that report the site of *dpp *gene transcription, and profile plots of the anti-pMad label intensity across the eye field were generated and compared with the source of Dpp (Figure 1G-K). The pMad levels increase gradually from low anterior levels to a peak that is anterior to *Dpp-LacZ *expression in the morphogenetic furrow. Within the morphogenetic furrow, pMad levels remained high although declining somewhat, then peaking again sharply at the posterior of the morphogenetic furrow around ommatidial column 3. Mad phosphorylation rapidly declined more posteriorly.

Double labeling with Cyclin B was performed to explore the relationship of Mad phosophorylation with the cell cycle (Figure 1L, M). The first peak of Mad phosphorylation occurred within the region of G1 arrest anterior to the furrow. The second peak corresponded to the second mitotic wave (SMW). Although the pseudo-stratification of the eye imaginal disc epithelium makes it difficult to measure the dimensions of the gradient precisely, there were about 30 nuclei between the two peaks of pMad (Figure 1S-U). Higher magnifications show that the second peak includes intense Mad phosphorylation of Cyclin B-positive cells that have re-entered the cell cycle. The pMad labelling of these cells appears to us qualitatively distinct, as though a different subcellular location is being labelled. Double-labeling with DRAQ-5 confirms that this is nuclear pMAD, however (Figure 1N-P).

To determine whether there was a polar-to-equatorial gradient of Mad phosphorylation, profile plots were compared at the equator (Figure 1J, M, R) and margins of the eye field (Figure 1V and data not shown). There was more Mad phosophorylation near the dorsal and ventral eye margins in the most anterior eye disc regions where levels were low overall, but no difference along the dorsoventral axis could be detected more posteriorly, Where pMad levels increased closer to the morphogenetic furrow.

Taken together, these observations suggest that most BMP activity in the third instar eye disc comes from the morphogenetic furrow, with polar Dpp expression making only a small contribution in anterior regions. The polar-equatorial BrkLacZ expression reported previously may reflect perdurance of beta-Galactosidase protein from an earlier developmental stage, prior to morphogenetic furrow initiation [16]. Most Mad phosphorylation occurs within the disc epithelium that is expressing Dpp, with activity in the overlying peripodial cell layer barely detectable.

### BMP regulation of the eye disc cell cycle

**Figure 2:**
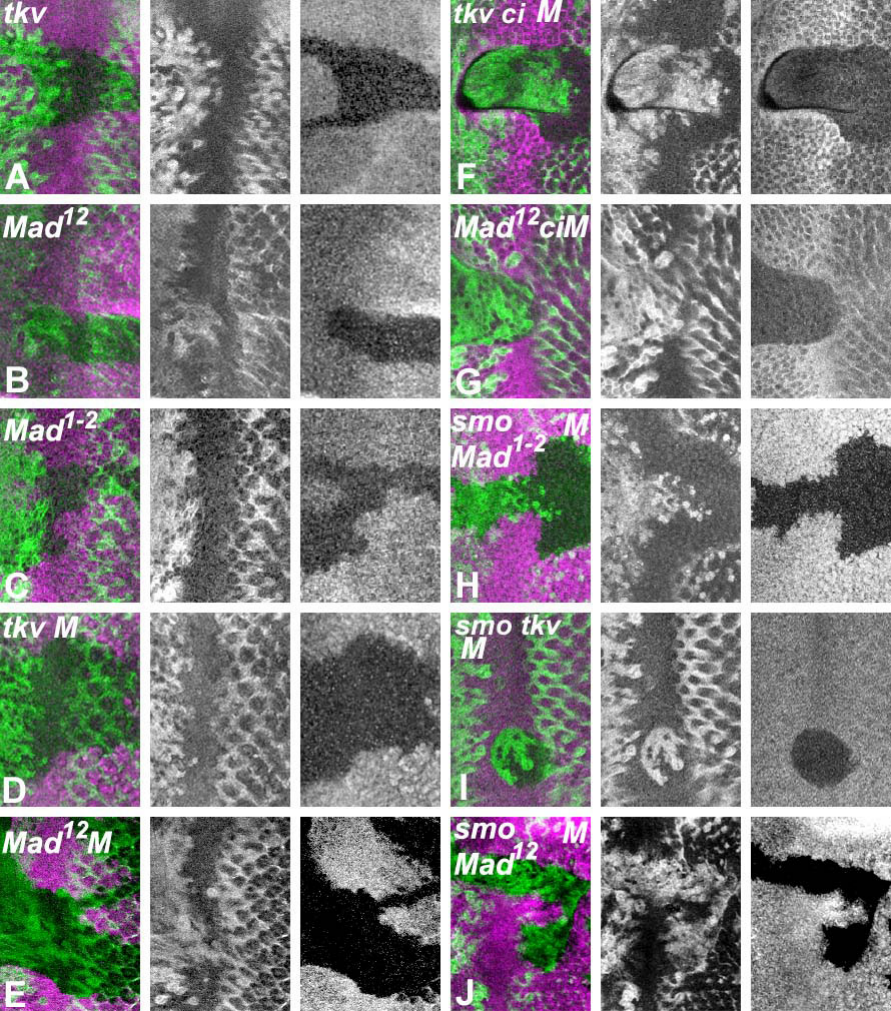
**G1 arrest requires Dpp and Hh signal reception**. All figures show Cyclin B protein (green) in mutant clones and neighboring wild type regions spanning the morphogenetic furrow, with anterior to the left. Homozygous cells are identified by the absence of β-galactosidase (magenta). (A) *tkv*^*4*^; (B)*Mad*^*12*^; (C) *Mad*^*1-2*^; (D) *tkv*^*4 *^in *tkv +/+ M *background; (E) *Mad*^*12 *^in *Mad +/+ M *background; (F) *tkv*^*4*^*ci*^*94 *^in *tkv ci +/+ + M *background; (G) *Mad*^*12*^*ci*^*94 *^in *Mad ci +/+ + M *background; (H) *smo Mad*^*1-2 *^in *smo Mad +/+ + M *background; (I) *smo*^*3*^*tkv*^*8 *^in *smo tkv +/+ + M *background; (J) *smo*^*3*^*Mad*^*12 *^in *smo Mad +/+ + M *background. Many of these genotypes have also been examined with anti-pH3 labelling to assess mitotic activity, and BrdU incorporation studies to measure S-phase DNA synthesis, confirming the results obtained with CycB labeling in all cases [26](data not shown).

**Figure 3:**
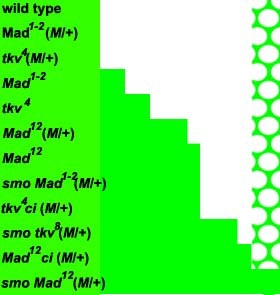
**Graded requirement for Dpp and Hh signal transduction**. This summary cartoon illustrates in the extent of cell cycle activity near the morphogenetic furrow in various genotypes. Green shading indicate regions where cells have progressed past the G1-S boundary, as indicated by CycB protein levels, for example, whereas white indicates regions where all cells remain in G1. In the case of wild type, shown at the top, all cells remain in G1 from anterior to the morphogenetic furrow until the Second Mitotic Wave starts, after which only the five cells of the photoreceptor preclusters remain in G1. These preclusters appear as the white circles in the Second Mitotic Wave pattern shown at the right of the diagram. Results from 11 other genotypes are summarized in order of severity, with the most severe at the bottom. In each case the approximate extent of the delay in G1 arrest that is observed in mutant clones is indicated. For simplicity, delays in the SMW that result from some genotypes are not included.

If there was a spatial gradient of Dpp concentration, hypomorphic mutations affecting the Dpp pathway should be insufficient for G1 arrest where Dpp levels were low, but would permit G1 arrest more posteriorly where Dpp levels were higher. It is informative to compare genotypes that affect Dpp signaling to different degrees. Some effects of mutations are shown in Figure 2, and all results summarized in Figure 3.

First, we compared cells mutant for *tkv*^*4*^, reported to be a null allele [22], the null allele *Mad*^*12*^, and the hypomorphic mutation *Mad*^*1-2*^. Cells homozygous for each genotype differed in cell cycle behavior. As described previously, cells homozygous for *tkv*^*4 *^arrested later than wild type cells, and so remained in G1 for a reduced period of time before either differentiating or re-entering the cell cycle in the Second Mitotic Wave that occurs posteriorly to the MF (Figure 2A) [8]. *Mad*^*12 *^clones were recovered rarely, only near the equator, and like *tkv*^*4 *^showed a significant delay in G1 arrest (Figure 2B). Clones of cells homozygous for *Mad*^*1-2 *^behaved more normally, sometimes showing a delay like that seen for *tkv*^*4*^, sometimes arrested in G1 only slightly later than wild type cells, and sometimes arrested indistinguishably from normal cells (Figure 2C, and data not shown). We did not notice any particular relationship between these outcomes and position in the anterior-posterior or polar-equatorial axes, or to the size of the *Mad*^*1-2 *^clones. *Mad*^*1-2 *^clones were obtained much more easily than clones of *tkv*^*4 *^cells or *Mad*^*12*^cells, were often larger, and were not restricted to the equatorial region as *tkv*^*4 *^or *Mad*^*12 *^clones were. These differences all support the notion that *Mad*^*1-2 *^affects BMP signaling less than *Mad*^*12 *^and *tkv*^*4 *^do.

Because cells mutant for *tkv*^*4 *^or *Mad*^*12 *^were rarely recovered near the equator, and not recovered elsewhere in the eye disc, we used the Minute technique to confer a competitive advantage on the mutant cells [23]. Larger *tkv*^*4 *^or *Mad*^*12 *^clones were obtained in the *M*/+ background. Strikingly, the cell cycle effect of *tkv*^*4 *^was suppressed in the *M*/+ background, as *tkv*^*4 *^homozygous cells arrested at the same time as neighboring *M*/+ cells (Figure 2D). The cell cycle phenotype of *Mad*^*1-2 *^mutant cells was also lost in the *M*/+ background (data not shown). The *Mad*^*12 *^clones behaved differently, and retained cell cycle defects in the *M*/+ background (Figure 2E). In control experiments, *M/+ *cells arrested at the same time as neighboring wild type cells (data not shown). These findings suggested that *M*/+ neighbors rescued BMP signaling in nearby *tkv*^*4 *^or *Mad*^*1-2 *^cells, but not *Mad*^*12 *^cells. We also observed rescue of cell cycle arrest in clones homozygous for *punt*^*135*^, a mutation in the Type II receptor chain, in a *M*/+ background (data not shown).

Cell competition may account for some of these results. It has been suggested that in the presence of wild type cells, *M*/+ cells compete less effectively for Dpp [24]. If this is correct, more Dpp could be available to *tkv*^*4 *^or *Mad*^*1-2 *^cells surrounded by *M*/+ cells, explaining their partial rescue. The finding that *Mad*^*12 *^cells were not rescued in the *M/+ *background suggests that an increase in available Dpp makes little difference to *Mad*^*12 *^cells. This interpretation implies that *tkv*^*4 *^cells are able to respond to Dpp when more is available. As mentioned above, the other Type I receptor chain encoded by *sax *could provide Tkv-independent signaling. In addition, although *tkv*^*4 *^is associated with a premature stop codon within the open reading frame, we suspect that *tkv*^*4*^is not null, because the *tkv*^*4 *^homozygous phenotype was suppressed by homozygosity for a R239C mutation in the EF1α-like factor, a translation termination factor (J. Curtiss and N. Zhuo, unpublished results). Such suppression is usually an indication that some translational read-through of a stop codon occurs, even in the presence of normal EF1α-like factor[25].

The experiments described so far have impaired BMP signaling to six distinct levels in *Mad*^*12*^, *Mad*^*1-2*^, *tkv*^*4*^, *Mad*^*12 *^in *M*/+, *Mad*^*1-2 *^in *M*/+, or *tkv*^*4 *^in *M*/+, affecting G1 arrest anterior to the MF to different degrees. Further changes in cell cycle behavior were observed when Hh signaling was also impaired[26]. A summary of the positions where G1 arrest occurs in cells with 12 different capacities to respond to BMP and Hh signaling is shown in Figure 3. Mutation of *smo*, the receptor for Hh, had little effect on G1 arrest in wild type cells but greatly retarded arrest in *tkv*^*4*^, *Mad*^*1-2 *^or *Mad*^*12 *^cells (Figures 2H-J and data not shown). The same phenotypic series was observed, so that *smo tkv*^*4 *^cells arrested later than *smo Mad*^*1-2*^, and *smo Mad*^*12 *^cells never arrested.

Our results indicate that normally, little Hh reaches the point of G1 arrest in wild type, but significant Hh reaches the locations where delayed G1 arrest occurs, and could potentially contribute to these arrests by reducing Ci75 levels. Absence of Ci protein also enhanced the G1 arrest defect of different Dpp pathway mutants. Loss of Ci had no effect on *Mad*^*1-2*^, and enhanced delays in *tkv *and *Mad *null cells less than *smo *mutations did (Figure 2F, G, and data not shown). Thus, the requirement of Hh increased as the level of Dpp signaling was reduced.

Taken together, these results show that requirements for BMP and Hh signaling are graded from anterior to posterior (Figure 3). These data are consistent with posterior to anterior gradients of Hh and BMP proteins that require BMP and Hh pathway components more stringently anteriorly in order to achieve a threshold response necessary for G1 arrest. However, the results are also consistent with an alternative possibility; perhaps it is sensitivity to BMP and Hh that varies, with higher levels being required by less sensitive anterior cells. In principle a graded response, rather than a gradient of Dpp activity, could also explain the observations.

### Induction of G1 arrest by activated Thickvein

If a threshold concentration of Dpp defines the onset of G1 arrest, then G1 arrest should occur earlier when Dpp signaling is elevated. If other genes define a graded requirement for Dpp signaling, then elevating Dpp signaling should not affect the position where G1 arrest occurs. To distinguish these models, we generated clones of cells expressing a constitutively active form of the Dpp Type I receptor, Tkv (*tkv*^*QD*^) [1] and positively marked them with GFP (*act>tkv*^*QD*^, *GFP*). The clones expressing Tkv^QD ^cell-autonomously elevated Mad phosophorylation levels higher than is seen in wild type eye discs (data not shown).

**Figure 4:**
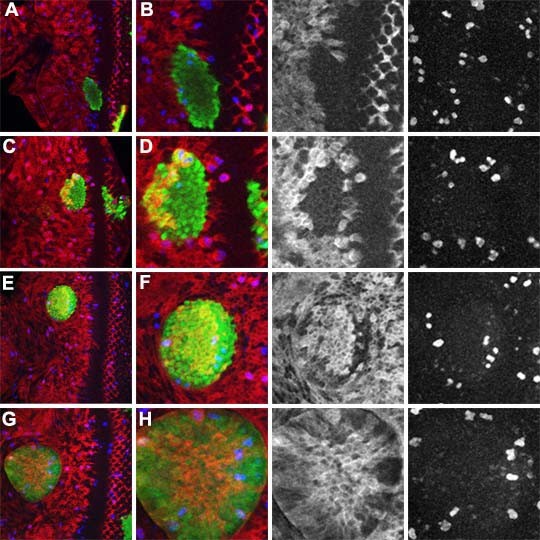
**Induction of G1 arrest by activated Thickvein**. The activated receptor Tkv^QD ^was expressed in clones also expressing GFP (green). Cell cycle activity was monitored through labeling for CycB (red) and phospho-H3 (blue). These two markers gave consistent results. 50 Tkv^QD^-expressing pH3-labelled cells in thirteen clones were CycB positive, while 17 pH3-labelled cells in anaphase/telophase were Cyclin B negative, reflecting the metaphase proteolysis of Cyclin B. (A) Near the morphogenetic furrow, close to where wild type cells arrest in G1, Tkv^QD ^accelerates arrest of all cells; (B) Higher magnification and separate channels; (C) Clones expressing Tkv^QD ^at more anterior locations continued proliferating before arresting about twice as far from the morphogenetic furrow as wild type cells, so that each clone had an anterior, proliferating segment and a posterior, arrested segment; (D) Higher magnification and separated channels; (E) Clone located more anteriorly than that in panel C; (F) higher magnification and separated channels; (G) In the most anterior parts of the eye disc, Tkv^QD^expression was not sufficient to cause cell cycle arrest; (H) higher magnification and separated channels.

The *act>tkv*^*QD *^clones were similar in size to control clones expressing GFP alone, indicating that Tkv^QD ^does not block all proliferation. Tkv^QD ^expressing cells arrested in G1 earlier (ie more anteriorly) as the furrow approached and were often already arrested while nearby wild type cells continued to cycle (Figure 4). The arrested regions lacked CycB expression or pH3-labeled mitotic figures (Figure 4). The results defined a boundary where *act>tkv*^*QD *^cells arrested in G1. This boundary lay about twice as far ahead of the furrow as the arrest of wild type cells (Figure 4). Clones of *act>tkv*^*QD *^cells spanning this boundary contained anterior, proliferating cells while the posterior of the clone had arrested (Figure 4). These findings indicate that elevated Dpp signaling induces G1 arrest earlier in development, within a domain close to the location where G1 arrest normally occurs, but does not prevent proliferation of cells in the very anterior eye, far from the morphogenetic furrow. This explains how *act>tkv*^*QD *^clones are found throughout the eye disc, since they can grow everywhere before morphogenetic furrow progression begins.

These observations show that activated Dpp signaling is sufficient to arrest the cell cycle in cells ahead of the furrow and strongly supports the idea of cell cycle arrest in response to a Dpp gradient. However, our findings further indicate that cells far anterior to the morphogenetic furrow are not yet competent to arrest in response to Dpp, and that low Dpp signaling is not the only factor in the continued proliferation of such anterior cells.

### Induction of cell cycle arrest by Dpp

**Figure 5:**
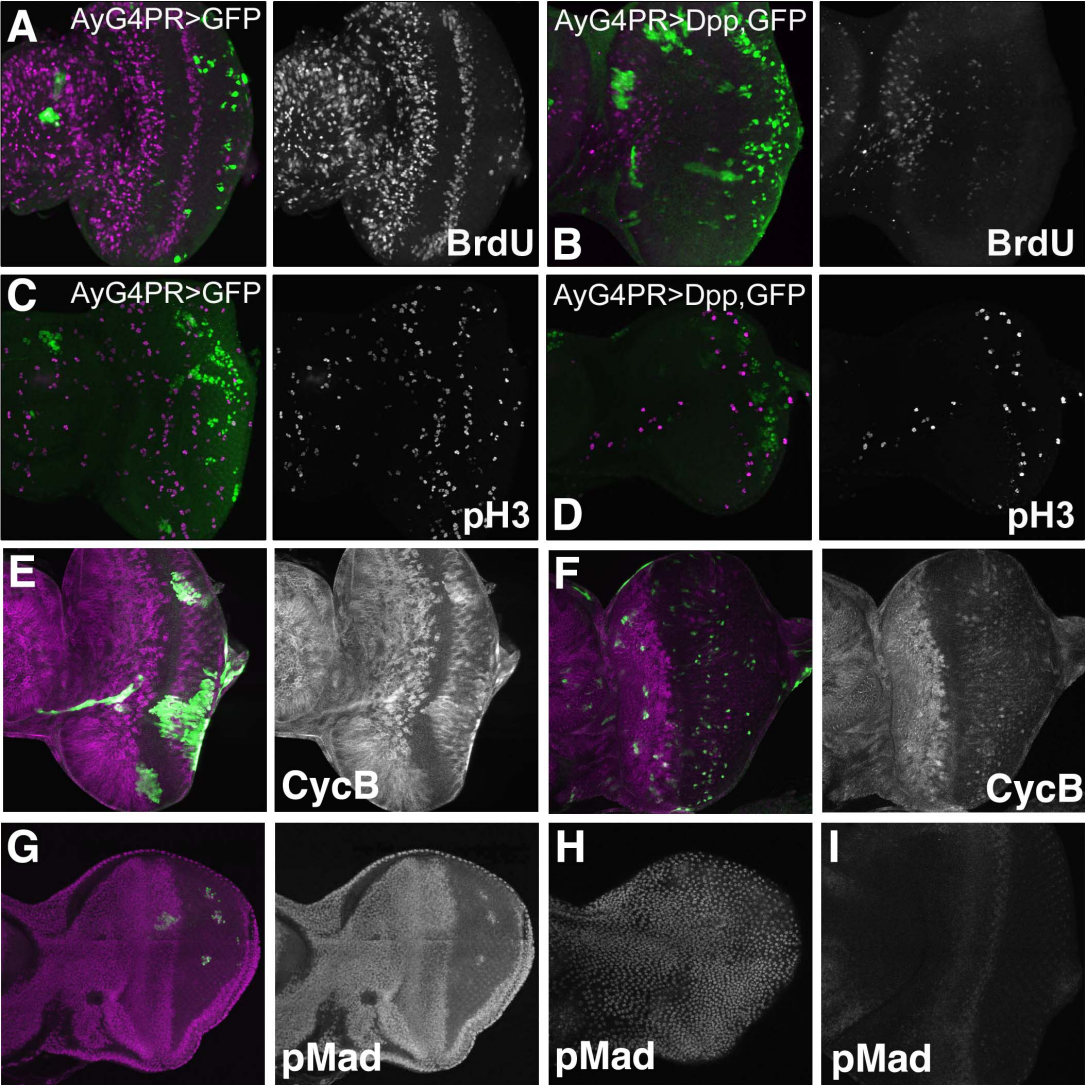
**Cell cycle arrest in response to ectopic Dpp**. (A) Inducible expression of GFP in clones did not affect cell proliferation in eye discs (BrdU incorporation in magenta); (B) Inducible expression of Dpp in clones almost completely eliminated BrdU incorporation (magenta) throughout the eye disc, antennal disc, and peripodial membrane; (C) Inducible expression of GFP in clones did not affect cell proliferation in eye discs (phospho-H3 labeling in magenta); (D) Inducible expression of Dpp in clones greatly reduced phospho-H3 labeling of mitotic figures; (E) Inducible expression of GFP in clones did not affect CycB expression in eye discs (magenta); (F) Inducible expression of Dpp in clones led to complete loss of CycB expression posterior to the morphogenetic furrow. Anterior to the position where cells normally arrest in G1, where wild type discs contain cells at varied cell cycle stages, ectopic Dpp led to accumulation of CycB in all the arrested cells. CycB in magenta; (G) Even a small proportion of cells expressing Dpp were sufficient for intense Mad phosphorylation (magenta) throughout much of the eye antennal disc. Posterior to the furrow, however, highest levels of pMad were only seen in the cells expressing Dpp themselves; (H) High pMad levels throughout the peripodial membrane of the disc also shown in panel G; (I) pMad labeling in a wild type eye disc processed and recorded in parallel to the disc in panels G and H.

Our findings differed from those described previously based on overexpression of Dpp itself[9]. Ectopic Dpp, expressed from a heat-inducible transgene, rapidly arrested cell proliferation throughout the eye disc, antennal disc, and peripodial epithelium[9], not only in a discrete portion of the eye disc epithelium as we found for ectopic Tkv^QD^. To explore this difference, we expressed ectopic Dpp using the clonal expression strategy. Despite not employing heat shock, the study confirmed the widespread effects reported by Horsfield et al (Figure 5).

The *act>dpp, GFP *clones were smaller than *act>GFP *clones induced in parallel, indicating an earlier or stronger inhibition of growth or survival than seen with Tkv^QD ^(Figure 5E, F). To assist clone recovery, a progesterone-inducible Gal4 method was used thereafter so that Dpp expression could be induced after clone growth had occurred[27]. Induction of Dpp secretion from such clones almost completely abolished BrdU incorporation in the entire eye-antennal disc and greatly reduced mitotic index (Figure 5A-D). Unexpectedly, CycB expression was affected differentially according to position. Cells arrested in the posterior eye or morphogenetic furrow region lacked Cyclin B expression, but cells arrested by Dpp in the anterior eye and in the antennal disc maintained high, uniform levels of CycB (Figure 5E-F). The latter indicates either arrest by Dpp at a cell cycle stage other than G1, or loss of cell-cycle regulation of Cyclin B expression and stability. These results represent a further difference from our findings with Tkv^QD^, and from what is seen in wild type development; in both these cases, Dpp signaling through Tkv arrested cells ahead of the furrow in G1, without CycB protein.

A small number of Dpp expressing cells was sufficient to phosphorylate Mad throughout the eye disc ahead of the furrow and antennal disc, to levels far higher than seen in wild type tissue (Figure 5G, I). This intense phosphorylation extended to the entire peripodial membrane, even in cases where the only Dpp secretion was in the disc epithelium (Figure 5H). These data imply activity of Dpp at extremely long range. Mad was less phosphorylated posterior to the furrow, although still to a level higher than seen in wild type development (Figure 5G, I). An exception to this was the cells posterior to the furrow that actually expressed Dpp; these phosphorylated Mad to a very high level in cell-autonomous fashion (Figure 5G).

It is surprising that ectopic Dpp produces effects not shown by activated Tkv, and implies that overexpressed Dpp has effects not mediated by Tkv, at least not by the activity that is activated in Tkv^QD^. One possibility is that ectopic, secreted Dpp acts indirectly ie that ectopic Dpp activates Tkv at some particular location in the eye antennal disc or elsewhere in the larva, stimulating another long-range signal that inhibits cell cycle progression through its own receptor. If this was so, some experiments expressing Tkv^QD ^in clones should have triggered the secondary non-autonomous signal, but we have yet to see global non-autonomous cell cycle arrest in response to Tkv^QD^. An alternative interpretation, which we favor, is that exceptionally high levels of ectopic Dpp have a non-physiological effect.

### Role of Homothorax

**Figure 6:**
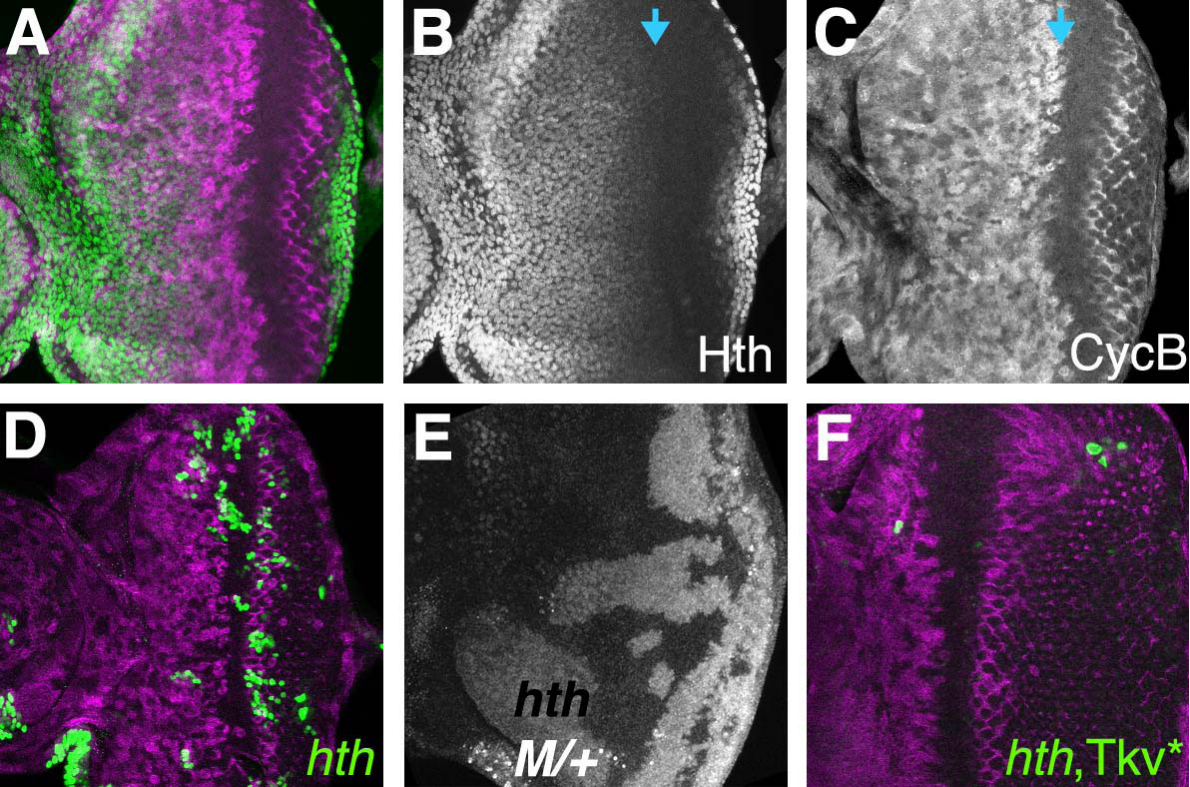
**Expression and requirement for *homothorax***. (A) Homothorax (green) is expressed in the anterior eye disc but repressed anterior to the morphogenetic furrow. Levels gradually reduce, starting while cells are still cycling (CycB in magenta); (B) Hth channel from panel A; Blue arrow shows onset of arrest, as measured by loss of Cyc B (C) CycB channel from panel A; (D) Clones of cells expressing GFP (green) and mutant for *hth *are recovered in posterior eye regions where cell proliferation stops earlier in development, but rarely recovered in the anterior eye. CycB in magenta; (E) Large *hth *mutant clones are recovered readily in a heterozygous Minute background. Clones identified by absence of beta-galactosidase labeling; (F) Clones of cells expressing both GFP (green) and Tkv^QD ^as well as mutant for *hth *are recovered very poorly in all locations of the eye disc. CycB in magenta.

It has been suggested that G1 arrest ahead of the furrow is due to loss of *hth *expression, because *hth *is required for proliferation and repressed by Dpp signaling, and because *hth *is homologous to vertebrate MEIS oncogenes[28]. We found that Hth expression starts to reduce before G1 arrest, defining a domain of cells that continue to proliferate while Hth expression drops (Figure 6A-C). Although *hth *mutant clones are difficult to recover in eye discs, they frequently survive in the posterior eye, where they can be rescued from competition by the more rapid arrival of the morphogenetic furrow (Figure 6D). Large *hth *mutant clones were recovered throughout the eye and antennal discs when the Minute technique was used (Figure 6E). We also found that whereas ectopic Tkv^QD ^usually represses eye disc Hth expression, the cell cycle is only arrested in a particular region (Figure 4). Together, these observations suggest that *hth *expression may not be essential for proliferation in the eye disc, and that *hth *repression is not sufficient to explain G1 arrest ahead of the furrow.

One possibility is that Hth expression defines the anterior cells that continue to proliferate regardless of Dpp signaling, and the cells that are sensitive to cell cycle arrest by Dpp are characterized by absence of Hth. To test this model, *act>tkv*^*QD *^clones that lack *hth *were generated using a MARCM method. Compared with *hth *clones induced in parallel experiments, *hth*, *act>tkv*^*QD*^clones were fewer in number and smaller (Figure 6D, F). This is consistent with the hypothesis that cells lacking *hth *expression are susceptible to cell cycle arrest in response to high Dpp levels. It was not possible to observe cell cycle arrest directly in *hth*, *act>tkv*^*QD *^clones, however, because such clones were so rare ahead of the furrow. We did not see an obvious cell cycle difference in *hth *clones in a M/+ background (Figure 6E).

## Discussion

We have investigated the potential role of a Dpp gradient in defining the anterior limit of the region of G1 arrest that precedes the morphogenetic furrow is defined. Our findings argue strongly that eye disc cells arrest in G1 at a particular threshold level of Dpp signaling, presumably in response to Dpp-regulated transcription of a gene or genes that regulate the cell cycle. We discuss several models for how this may be achieved, and suggest that the *hth *gene may play a role in defining the response to Dpp.

### There is a gradient of Dpp signaling derived from the morphogenetic furrow

The expression of Brk and Dad, and the phosphorylation status of Mad, confirm that Dpp signaling in the anterior eye disc centers on the morphogenetic furrow, where a stripe of Dpp transcription occurs. A gradient of Mad phosphorylation is oriented mostly posterior-to anterior ahead of the morphogenetic furrow. Most Dpp diffusion seems to occur within the plane of the epithelium, unless Dpp is over-expressed.

Unexpectedly, highest pMad levels occur in dividing cells just posterior to where Dpp is transcribed (Figure 1G-J). Although it has been proposed that Dpp is required for the Second Mitotic Wave [19], multiple studies describe normal Second Mitotic Wave divisions in cells mutant for the Dpp pathway[8,9,26]. Another possible explanation is that concentration of pMad in these cells is related to inactivation of Dpp responses posterior to the furrow.

### There is a graded requirement of BMP signaling components in cell cycle arrest arrest anterior to the MF

Mutations that limit the ability of a cell to respond to Dpp signals delayed the G1 arrest proportionately, so that G1 arrest occurred very late in cells that had little or no ability to respond to Dpp. The delay in G1 arrest could be rescued in a Minute background, and was enhanced when cells were also unable to respond to Hh signaling, further revealing quantitative differences between Dpp pathway mutations. Comparing many genotypes indicates that cells further from the source of Dpp transcription have a greater requirement for intact Dpp signal transduction. The graded requirement for Dpp signaling correlates well with the actual cellular levels of Dpp signaling activity; cells that have the highest requirement for Dpp signaling components are those towards the anterior which actually exhibit lower levels of Mad phosphorylation.

### A threshold level of Dpp signaling is responsible for cell cycle arrest

Constitutively activating Dpp signaling by expressing activated Tkv enlarged the domain of arrested cells (Figure 4), supporting the model that the level of Dpp signaling determines where arrest occurs within a competent domain anterior to the furrow. Further anterior, however, cells were not arrested by constitutive signaling. This difference helps understand how Dpp signaling can cause cell cycle arrest and also be required for the growth and survival of cells. We infer that Dpp is required for growth and survival in the early eye disc; closer to the furrow, Dpp signaling becomes sufficient to arrest the cell cycle.

Studies of wing development show that both the level of Dpp signaling and its gradient can affect growth and proliferation[27]. In some wing regions, growth occurs where nearby cells differ in Dpp signaling level, and not where nearby cells share similar signaling levels. Similar experiments using short term, progesterone-inducible expression of Tkv^QD ^confirm that discontinuities of Dpp signaling level induce growth in the anterior eye as in the wing (unpublished results). We think that cell cycle arrest close to the furrow is not explained simply by uniform high Dpp signaling, however, because cell cycle progression continues in cells that lack Dpp signaling, and because uniform expression of activated Tkv did not arrest the cell cycle in these same anterior eye regions. The results are more simply explained by G1 arrest being induced by a threshold of Dpp signaling level.

Different thresholds for regulation by Dpp signaling may also contribute to gene expression patterns ahead of the furrow. Gene expression patterns are complicated, however, because combinatorial regulation by other signals in addition to Dpp seems to be the rule. For example, the proneural bHLH gene *atonal (ato) *is only efficiently induced by Dpp where N signaling is also active [29,30], the retinal determination genes *eyes absent *and *dachshund *are turned on ahead of the furrow by both Dpp and Hh [31], the repression of *hth *depends on Ras signaling as well as Dpp [31], and the *hairy *gene is regulated by unknown signals in addition to Dpp (our unpublished results).

### The mechanisms of cell cycle regulation

Because the role of Dpp signaling in cell cycle control depends on *Mad*, it is thought to involve transcriptional regulation. A similar conclusion applies for Hh signaling [10].

It has been suggested that Dpp does not induce cell cycle arrest directly, but paradoxically does so indirectly by first promoting cell cycle progression [8,32]. The idea is that if Dpp accelerates the cell cycle, this will accelerate G1 arrest by cells that have received a distinct arrest signal but are not yet in G1. Evidence for this view comes from the modest increase in mitotic activity that is sometimes observed just anterior to the G1 arrest, the so-called 'first mitotic wave'[6]. Cells mutant for Dpp signaling components show less mitotic activity in this region, consistent with induction of the first mitotic wave by increasing Dpp signaling[32](our unpublished results).

Our model for Dpp raises an alternative possibility. If Dpp promotes cellular growth in the anterior region of the eye disc, this may contribute to mitotic activity throughout the anterior, proliferating region. Cells lacking this input would be expected to divide less in the 'first mitotic wave', as well as at other locations in the anterior eye disc. Further studies will be required to distinguish these possibilities.

The *hth *gene, which encodes a transcription factor homologous to the MEIS family of proto-oncogenes, is a potential explanation for the changing response to Dpp. It has been suggested before that *hth *is required for cell cycle progression, and that repression of *hth *expression by Dpp leads to cell cycle arrest[28]. Our data suggests a modification of this model. We propose that *hth *protects cells from cell cycle arrest in response to Dpp, so that cells lacking *hth *are prone to cell cycle arrest and therefore unable to grow. One complication of our model is that *hth *expression is itself repressed by Dpp, raising the question of why activated Dpp signaling is not always sufficient to arrest the cell cycle, after first repressing *hth*.

Another recent suggestion is that Dpp and Hh are not sufficient to account for G1 arrest, because a vestigial arrest remains in clones of *smo*^*3*^*tkv*^*4 *^cells [32]. Comparing our extensive set of mutant genotypes strongly suggests that *tkv*^*4 *^mutation is not null, and that even *tkv*^*8*^, a more likely null mutation, may not eliminate Dpp signaling as completely as *Mad *mutations do (Figure 3). Residual Dpp signaling may therefore be responsible for the limited G1 arrest observed in *smo*^*3*^*tkv*^*4 *^cells. Escudero et al also observe G1 arrest in *Mad*^*12 *^*ci *cells, contradicting our previous findings [26]. Here we study a further genotype that completely lacks Dpp and Hh signaling, *Mad*^*12 *^*smo*^*3*^, and confirm that such cells do not arrest in G1 (Figure 2J).

A second argument for additional arrest signals has been that cells in the posterior part of the morphogenetic furrow remain arrested following ectopic expression of CycE or of E2F[32]. These observations, which are similar to some that have been made previously [33], could also be explained by the breakdown in the positive feedback between CycE/Cdk2 and E2F1 activities that occurs as differentiation approaches, so that activation of both becomes required to drive cell cycle entry [26,34].

Clearly, further work will be required to fully unravel the mechanisms of cell cycle arrest at the molecular level. Although the possibility exists that signals besides Dpp and Hh are involved, in our view the evidence for such signals is not compelling at present.

## Conclusions

Our studies establish that a gradient of Dpp signaling, mostly directed along the anterior-posterior axis from the morphogenetic furrow, triggers the G1 arrest that precedes the onset of differentiation when a particular threshold of Dpp signaling activity is reached. Dpp signaling is only sufficient for cell cycle arrest within a portion of the eye disc, however, and the most anterior regions are insensitive. This permits Dpp to promote eye disc growth through much of development, only triggering cell cycle arrest as the morphogenetic furrow approaches in the late third instar. It was previously suggested that G1 arrest occurs when *hth *expression is downregulated, but our data suggests that *hth *downregulation only defines the competence for this response to Dpp. Our study also addresses outstanding questions concerning whether other spatial signals in addition to Dpp and Hh regulate G1 arrest, discrepancies between different methods to activate Dpp signaling, and the extent to which Dpp signals between disc epithelium and peripodial epithelium.

## Methods

### Mitotic Clone Induction

Clones of cells mutant for genes were obtained by the FLP-mediated mitotic recombination technique [35,36]. Homozygous mutant cells were identified through lack of Ci^*155 *^antibody staining, or the absence of transgene encoded marker arm-βgal.

*thickvein (tkv) *clones were obtained in hsF; *tkv*^*4 *^FRT40/[*armLacZ*] FRT40 and hsF; *tkv*^*4 *^FRT40/M(2)24F [*armLacZ*] FRT40 larvae as described [37]. *tkv*^*4 *^is reportedly a null allele[22]. *Mothers against dpp (Mad) *clones were obtained in hsF; *Mad*^*1-2 *^FRT40/[*armLacZ*] FRT40, hsF; *Mad*^*12 *^FRT40/[*armLacZ*] FRT40 and hsF; *Mad*^*12 *^FRT40/M(2)24F [*armLacZ*] FRT40. *Mad*^*1-2 *^is an insertion in Mad regulatory sequences that prevents most Dpp signaling with little effect on growth[38]. *Mad*^*12 *^is a null allele that lacks the N-terminal sequences for phosphorylation by BMP family receptors [39,40]. *smo Mad c*lones were obtained in hsF; *smo*^*3 *^*Mad*^*1-2 *^FRT40/M(2)24F [*armLacZ*] FRT40 larvae and hsF; *smo*^*3 *^*Mad*^*12 *^FRT40/M(2)24F [*armLacZ*] FRT40. *smo tkv c*lones were obtained in hsF; *smo*^*3*^*tkv*^*8 *^FRT40/M(2)24F [*armLacZ*] FRT40 larvae. *tkv*^*8 *^is a null allele [41]. *smo ci c*lones were obtained in *y *hsF; *smo*^*3 *^FRT40/[*ci*^*+*^] FRT40; *ci*^*94 *^larvae. The [ci+] transgene was provided by R. Holmgren. *Mad ci *clones were obtained in *y w *hsF; *Mad*^*1-2*^FRT40/[*ci*^*+*^] FRT40; *ci*^*94 *^and *y w *hsF; *Mad*^*12 *^FRT40/M(2)24F [*ci*^*+*^] FRT40; *ci*^*94 *^larvae. *tkv ci *clones were obtained in *y w *hsF; *tkv*^*4 *^FRT40/M(2)24F [*ci*^*+*^] FRT40; *ci*^*94 *^larvae. *Minute (M) c*lones were obtained in hsF; M(2)24F [*armLacZ*] FRT40/FRT40 larvae. - *homothorax *(*hth*) clones were obtained in hsF; FRT82 *hth*^*P2*^/FRT82 M [*armLacZ*]. Tkv^*QD *^was over-expressed in *hth*^*P2 *^clones using the MARCM technique [42]; *y w hsFLP, UAS-GFP/+*; *ActGal4*/*UAS-tkv*^*QD*^; FRT82 *hth*^*P2*^/FRT82 *TubGal80 and y w hsFLP, UAS-GFP/+*; *ActGal4*/+; FRT82 *hth*^*P2*^/FRT82 *TubGal80 *were simultaneously heat shocked at 24-72 hrs AEL and dissected at wandering third instar. The MARCM clones were positively marked and detected with an antibody against GFP.

### Flp-out Clone Induction

Constitutive flp-out clones of activated Tkv (Tkv^*Q*253*D*^) or full-length Dpp Dpp were generated by crossing *w hs-Flp*^*122*^; *UAS-tkv*^*QD*^*/TM6B *and *w hs-Flp*^*122*^; *UAS-Dpp/TM6B *to *y w p [act>CD2>Gal4]; UAS-GFP*. Adults were removed 24-48 hours AEL and larvae were heat shocked at 37°Non-*Tb *females were dissected 2 days later. Female *Tb *larvae from the same cross were used as control. Clones were detected with an antibody against β-Galactosidase.

Inducible flp-out Tkv* and Dpp clones were generated using the RU486 induced Gal4 [27], pAyGal4:PR. *w hs-Flp*^*122*^*;UAS-tkv*^*Q*253*D*^*/TM6B *and *w hs-Flp*^*122*^*; UAS-dpp*/TM6B were crossed to *UAS-GFP; pAyGal4:PR/TM6B*. Flp-out clones were then generated by heat shock for 7 min at 36°C. Gal4:PR was activated at a high dose as described for the length of time stated in the figure legend. *Tb *larvae from the same cross were used as control. Clones were detected with an antibody against GFP.

### Drosophila strains

Dad LacZ [42]; *UAS-dpp *[43]; *UAS-tkv*^*Q*253*D *^[1]; *act>CD2>Gal4 *[44]; *UAS-GFP; pAyGal4:PR/TM6B *[27]

### Tissue Staining and Immunofluorescence

Labeling of eye discs and BrdU incorporation was performed as described in [45]. Preparations were examined on the BioRad MRC600 Confocal microscope. Images were processed using Adobe Photoshop 4.0 and NIH Image J software. The signal plot of pMad was performed in Image J with a Gaussian blur of 2 px. Primary antibodies used were anti-Brk [14]; rat anti-Ci^155 ^(mAb2A1) [46]; anti-phospho-Smad1 [47]; anti-Cyclin B (F2F4) [48]; rabbit anti-β-Galactosidase (Cappel), mouse anti-β-Galactosidase (401a) (Developmental Studies Hybridoma Bank); mouse anti-BrdU (Becton Dickinson); mouse and rabbit anti-GFP antibodies (Invitrogen #A11120 and A11122); rabbit anti-phosphoHistone3 (Cell Signaling Technology #9701). To visualize the nuclei, Draq5 (Alexis Biochemicals, BOS-889-001-R200) was added to each of the detergent based washes after incubation in the secondary antibody at a final concentration of 500 μM.

## Authors' contributions

LCF carried out most of the experimental studies and drafted the manuscript. AB carried out the activated-Tkv mis-expression study. NEB conducted some of the experimental studies and participated in the design and coordination of the study as well as editing and revising the manuscript. All authors read and approved the final manuscript.
